# Supplementary material for: Early Motor Repertoire of Very Preterm Infants and Relationships with 2-Year Neurodevelopment
Source: J Clin Med. 2022 Mar 25;11(7):1833. doi: 10.3390/jcm11071833 (PMC9000187; doi:10.3390/jcm11071833)
Supplement: Supplementary file 1 [file jcm-11-01833-s001.zip › jcm-1617092-supplementary.pdf]

Table S1. Perinatal characteristics of infants with incomplete data

Perinatal details	MOS-R and 2-year follow-up completed	2-year follow up incomplete	p-value
	N=169	N=119	
Gestational age at birth weeks, mean (SD) *n*	28.6 (1.9)	28 (2) 113	0.35
Birth weight g, mean (SD) *n*	1097 (310)	1107 (304) 113	0.78
Males	95 (56%)	61/114 (54%)	0.71
Multiple births	54 (32%)	25/113 (22%)	0.079
Higher social risk	81/168 (48%)	65/94 (69%)	0.001
IVH grade 3 or 4	16 (9%)	6/82 (7%)	0.64
Periventricular leukomalacia	8 (5%)	3/71 (4%)	1.00
Postnatal corticosteroids	30 (18%)	5/72 (7%)	0.029
Bronchopulmonary dysplasia	65 (38%)	29/72 (40%)	0.89
3-4 month assessment			
Corrected age of GMA/MOS-R assessment, weeks, mean (SD) *n*	13.4 (1.3)	13.4 (1.4) 40	0.94
MOS-R, median (IQR) *n*	24.0 (21.0-26.0)	24.0 (21.0-26.0) 53	0.68
GMA			
Normal	154 (91%)	49/52 (94%)	
Abnormal	1 (0.6%)	0/52 (0%)	0.68
Absent	14 (8%)	3/52 (6%)	

p-values obtained using Fisher's exact test (categorical) or t-test (continuous) comparisons with infants with complete data.

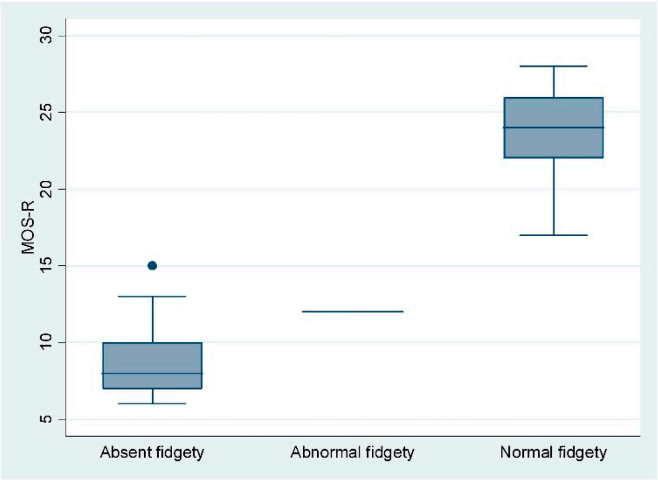

Figure S1. Box and whisker plot of MOS-R scores by GM score category (Absent, Abnormal, Normal fidgety)
